# Investigating the encrustation of reinforced ureteral stents by computational flow dynamic simulations

**DOI:** 10.1007/s00345-023-04356-5

**Published:** 2023-03-17

**Authors:** Benoît Vogt

**Affiliations:** Department of Urology, Polyclinique de Blois, 1 Rue Robert Debré, 41260 La Chaussée Saint-Victor, France

**Keywords:** Mathematical modeling, Computational fluid dynamics, Ureteral stent, Encrustation, Peristaltic movement, Stent failure

## Abstract

**Purpose:**

In cases of extrinsic ureteral obstruction, obstruction due to encrustation is particularly detrimental to functioning of the stent. A thorough understanding of the causes that lead to stent encrustation is essential. Computational fluid dynamic (CFD) simulations may provide a reliable screening platform for investigating the interplay between flow processes and encrustation dynamics in stents.

**Methods:**

Using a tailor-made program, we attempted to evaluate a number of reinforced ureteral stents by CFD simulations with an obstructed or unobstructed ureter and steady or discontinuous flow patterns to identify critical regions with abrupt changes in shape susceptible to stagnant flow and encrustation.

**Results:**

For the Vortek^®^ and Urosoft stents, the longitudinal opening of the stents confirmed the presence of critical regions. No critical region was observed for the Superglide stent. CFD simulations showed that cavities formed near the critical regions represented patently stagnant flow and were potentially susceptible to the formation of encrusting deposits. Encrustations were greater in the obstructed design than in the unobstructed design. In the model with a suddenly interrupted laminar flow, the peristaltic motion resulted in new discontinuous encrustation areas scattered throughout the entire external and internal surface of the stent.

**Conclusion:**

The analysis of fluid dynamics through the tested stents confirmed that encrustations are possible in regions of stagnant flow and showed that stent models with the smoothest possible surface are preferable. The discontinuous flow model provided results that are closer to the findings observed in the clinic and should be more often integrated into CFD simulations.

**Supplementary Information:**

The online version contains supplementary material available at 10.1007/s00345-023-04356-5.

## Introduction

The causes of extrinsic ureteral obstruction (EUO) requiring an indwelling stent are often benign or malignant extrinsic strictures (tumor compression or, post-radiation or post-surgical damage). However, ureteral obstruction is a challenge in the management of stent patency and most studies report an approximately 28% failure rate. Stent failure can induce renal failure, renal colic, or pyelonephritis [1–4].

The phenomenon of stent failure in the context of EUO is commonly attributed to deformation of the ureter and external pressure, which leads to occlusion of the stent lumen. Nevertheless, the reality appears to be more complex. Shilo et al. suggested that it is the combination of external pressure, deformation, and colloid concentration that leads to stent failure. In EUO, urine flows exclusively through the stent lumen without any possibility of passage through the extraluminal space between the stent and the ureter wall [5]*.* Thus, maintaining flow through the stent lumen is an essential parameter to preserve the flow rate, but it can be altered by encrustation.

Bacterial infection is known to be an important factor in the development of encrustations in urinary stents [6, 7]*.* It is well established that hydrodynamic forces play a crucial role in bacterial attachment and De Grazia et al. suggested that one of the factors that govern bacterial attachment in a stented ureter is the presence of cavity flow in areas located near a ureteral obstruction [8]. Thus, it is essential to identify critical regions susceptible to stagnant flow because increasing flow rates in these critical regions, by locally altering the architecture of the stent, could promote fluid drainage through the stent [5, 8, 9].

In previous studies, several reinforced ureteral stents were physically and clinically tested in patients [3, 4, 10]*.* The present study attempted to evaluate these same stents by computational fluid dynamic (CFD) simulations to identify critical regions susceptible to stagnant flow.

## Materials and methods

A number of reinforced double-pigtail stents without holes, such as the Vortek^®^ Tumor Stent (7F, Coloplast, Denmark), Urosoft Tumor Stent (7F and 8F, Bard Angiomed, Germany), and Superglide Tumour DD Ureter Stent (8F, Teleflex Medical, Ireland) have been physically (stiffness, lumen) [4, 10] and clinically (stent failure, survival) [3, 4] evaluated in previous studies. The geometry of each stent was determined after opening them along the longitudinal axis. The analysis focused on the internal structure of the stent, with the identification of abrupt changes in caliber or the shape of the internal surface.

**Fig. 1:**
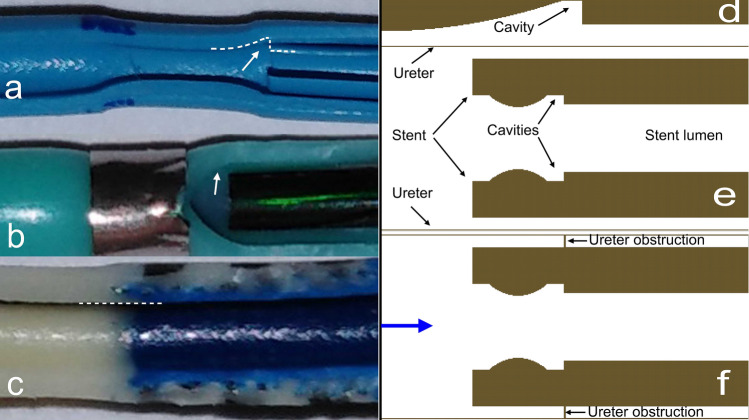
Reinforced double-pigtail stents opened along the longitudinal axis and computational meshes with quadrilateral elements for calculation of the governing equations. **a** Vortek^®^ Tumor Stent (7F, Coloplast, Denmark). The arrow and dotted line show the cavity between the tubular constriction zone and the sleeve. **b** Urosoft Tumor Stent (8F, Bard Angiomed, Germany). The arrow shows the same observations as in (**a**). **c** Superglide Tumour DD Ureter Stent (8F, Teleflex Medical, Ireland). The dotted line shows that the junction between the white loop and the blue reinforced tube is smooth and regular. **d** Mesh 1 focused on the stent cavity that was observed after opening the stent along the longitudinal axis. **e** Mesh 2 represented the longitudinal section of the stent within the ureter, focusing on the observed differences in the shape of the surface. **f** Mesh 3 was designed to replicate the geometric features of **e** with an obstruction. The blue arrow indicates the inlet flow direction

Two-dimensional numerical meshes were developed and computational fluid dynamic (CFD) simulations were used to further investigate urine flow dynamics near abrupt changes in shape of the internal surface of the ureteral stents. Three meshes were designed to replicate the geometric features of a stented ureter in the presence of different types of ureteral stents (Vortek^®^ or Urosoft stents) (Fig. 1).

The Navier–Stokes equations describe compressible and non-compressible flow and are the starting point for CFD calculations. To facilitate the obtention of a numerical solution, it is necessary to change the form of Navier–Stokes equations from partial differential equations to algebraic equations by a process called discretization [11]. Temporal discretization of the Navier–Stokes equations for non-compressible flow in vector form for a velocity field $\overrightarrow{v}$ can be written as Eq. (1), where *p, η, μ*, $\overrightarrow{\mathrm{grad}}$, $\overrightarrow{\Delta }$, $\overrightarrow{f}$*, t,* and Δt represent the pressure, dynamic viscosity, density, gradient, Laplacian vector, volume forces on fluid particles, time, and time-step, respectively.

1 $$\frac{{\overrightarrow{v}}^{t+1}-{\overrightarrow{v}}^{t}}{\Delta t}=-\left({\overrightarrow{v}}^{t}.\overrightarrow{\mathrm{grad}}\right) {\overrightarrow{v}}^{t}- \frac{1}{\mu } \overrightarrow{\mathrm{grad }}\left({p}^{t}\right)+ \frac{\eta }{\mu } \overrightarrow{\Delta }{\overrightarrow{v}}^{t}+\overrightarrow{f}$$

The equations and intermediate calculations are presented in the Online Resource 1. The model design is summarized in a movie in Online Resource 2 from governing equations to simulations of urine flow dynamics and encrustations.

## Results

For the Vortek^®^ and Urosoft stents, the longitudinal opening of the stents confirmed the abrupt change in shape previously suggested during the physical analysis of the stents [10] and showed cavities between tubular constriction zones and the sleeve. No changes in shape were observed for the Superglide stent (Fig. 1).

In the case of steady laminar flow, fluid flow was fastest at the center (0.7 mm s^−1^) and slowest close to the stent wall (velocity of approximately 0.1 mm s^−1^ and wall shear stress (WSS) < 70 mPa).

**Fig. 2:**
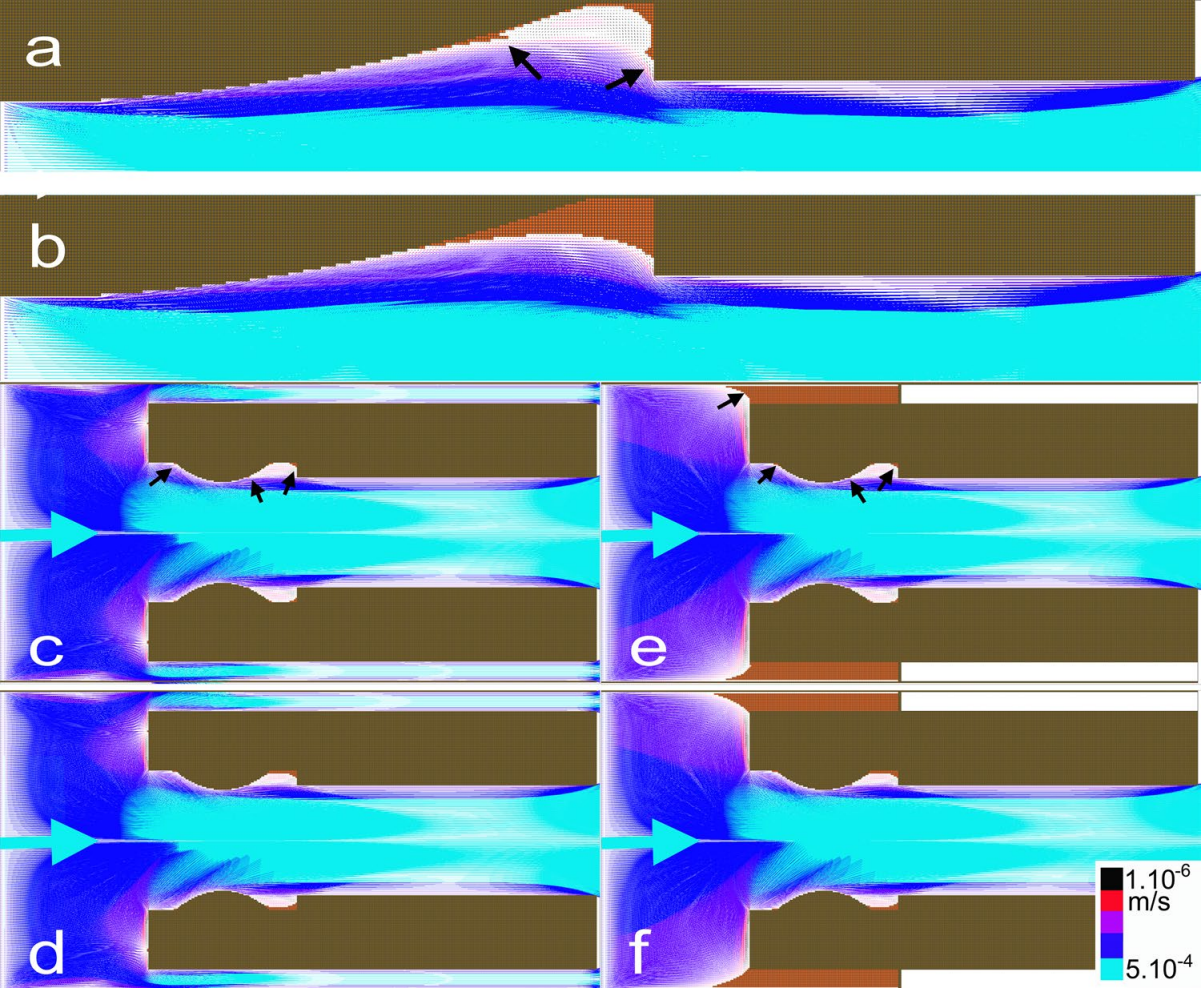
Flow vectors determined from CFD simulations for a stented ureter with a Vortek^®^ stent. **a** Flow vectors determined from CFD simulations for Mesh 1. CFD simulations showed very low WSS within the cavity. This region was also characterized by the presence of a laminar vortex. After temporal iterations and obtaining a steady state, encrusting deposits were observed in the cavity (black arrows). **b** After updating Mesh 1 with the new encrustations, the equations were used to calculate subsequent solutions until a steady state was achieved for which no WSS value was below 1 mPa. The areas of encrusted deposits then appeared to be more extensive. **c** Flow vectors determined from CFD simulations for Mesh 2. After temporal iterations and obtaining a steady state, encrusting deposits were observed close to the curvatures and cavities (black arrows). **d** After updating Mesh 2 with the new encrustations and obtaining a steady state, the areas of encrusted deposits then appeared to be more extensive. **e** Flow vectors determined from CFD simulations for Mesh 3. After temporal iterations and obtaining a steady state, encrusting deposits were observed close to the curvatures and cavities and led to clogging of all the cavity areas between the stent and ureter (black arrows). **f** After updating Mesh 3 with the new encrustations and obtaining a steady state, the areas of encrusted deposits then appeared to be more extensive. Legends: Results were obtained at a fixed volumetric flow rate of 0.5 ml min^−1^. The blue arrow indicates the inlet flow direction. The blue and red colors correspond to high and low velocity, as indicated in the color scale bar. Brown quadrilateral elements correspond to the regions of interest for which the WSS values were below 1 mPa and potentially susceptible to the formation of encrusting deposits

The cavity formed near the change in shape (Fig. 2a) represented a patently stagnant flow region characterized by a low velocity of approximately 0.001 mm s^−1^, a low WSS < 0.7 mPa, and the presence of a laminar vortex.

In the study, it is, therefore, anticipated that regions with a WSS < 1 mPa are potentially susceptible to the formation and growth of encrusting deposits in the stents. When the WSS is above 1 mPa, the formation of encrusting deposits in the stent is shown by brown quadrilateral elements in Fig. 2.

After updating the mesh with the new encrustations, the growth of encrusting deposits in the stent stopped after 13 iterations due to the WSS reaching more than 1 mPa (Fig. 2b). No encrustation was observed for the Superglide stent. In this geometry, CFD simulations showed the WSS to be consistently above 30 mPa close to the stent walls.

Numerical simulations showed the cavities formed by a ureteral obstruction to be characterized by a wide area of low WSS (low velocity and WSS < 1 mPa) (Fig. 2e). Encrustations were greater in the obstructed design than in the unobstructed design and led to clogging throughout the cavity area.

**Fig. 3:**
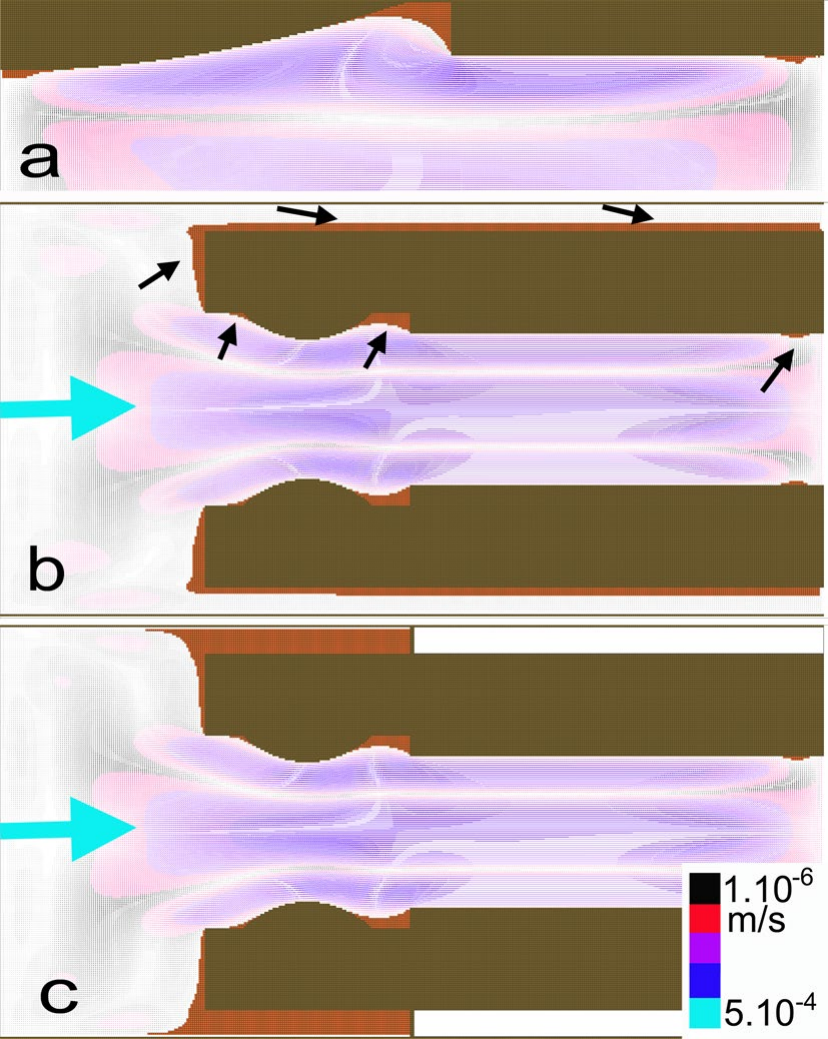
Flow vectors determined from CFD simulations for a stented ureter with a Vortek^®^ stent with an abruptly interrupted laminar flow to mimic pelvi-ureteric peristalsis. **a** Mesh 1 focused on the stent cavity. **b** Mesh 2 represented the longitudinal section of the stent within the ureter. **c** Mesh 3 was designed to replicate the geometric features of **b** with an obstruction. After temporal iterations, simulations highlighted the presence of new discontinuous encrustation areas scattered throughout the entire external and internal surface of the stent (black arrows). In this model, no steady-state solution was obtained because the encrusted mesh modification systematically resulted in a WSS < 1 mPa. The legends are the same as in Fig. 2

In the second model with an abruptly interrupted laminar flow, the peristaltic motion resulted in recirculation zones with negative velocities developing in the different parts of the ureter. Encrustation simulations highlighted the presence of new discontinuous encrustation areas scattered throughout the entire external and internal surface of the stent (Fig. 3). In this model, no steady-state solution was obtained because the encrusted mesh modification systematically resulted in a WSS < 1 mPa.

## Discussion

In cases of EUO, obstruction due to encrustation is particularly detrimental to functioning of the stent, as urine flows exclusively through the stent lumen without any possibility of passage through the extraluminal space [5]*.* A thorough understanding of the causes that lead to stent encrustation is essential. CFD simulation models provide a rapid, cost-effective, and reliable screening platform for investigating the interplay between flow processes and encrustation dynamics in stents. Thus, CFD simulations could be used to further advise stent development and assist in the selection of the most effective stents for drainage. [5, 8, 9, 11–14].

The mathematical solution used for this study was more rudimentary than the generally used software (such as Fluent^®^ (Ansys Inc., USA)). However, it incorporated the same approximations, and the results are consistent with those published in the field. Indeed, by running this solution on models previously presented by other authors (Fig. Online Resource 3), vortices, encrustations, and the same differences in WSS were noted, with rates of 40 mPa along the tube and 0.2 mPa in the cavities [8, 9, 12, 13]. In addition, simulations using meshes with different formats (100 × 800) with different critical values of encrustation (WSS = 2 to 20 mPa) and continuous or discontinuous flow confirmed the results presented in this study.

For the Vortek^®^ and Urosoft stents, the longitudinal opening of the stents confirmed the abrupt changes in shape previously suggested [10] and showed cavities. It is well established that the stent lumen is an important factor for increasing the volumetric flow rate [4, 10, 15]*.*

Alterations of the internal lumen of the Vortek^®^ and Urosoft stents may affect urine flow and lead to encrustations. Indeed, for laminar flow, quantitative analysis of colloid suspensions in tubular flow showed that colloids can accumulate particularly in regions where curvature, deformation, and compression alter the cylindrical cross-section of a tube [5]*.* However, no encrustation was seen for the Superglide stent. In this geometry, the stent design presented no visible attachment, suggesting that the WSS was not sufficiently low to initiate encrustations. Based on a control stented ureter system, the experiment of Shilo et al. found that colloids alone do not cause stent failure over time. Rather, it was the combination of external pressure, deformation, and colloid concentration that can lead to encrustations and stent failure [5]*.*

As shown in Fig. 2, the cavities, where low-velocity laminar vortices were observed, are characterized by a low WSS < 5 mPa. It was, therefore, anticipated that these regions were potentially susceptible to the formation and growth of encrusting deposits.

These results are in accordance with those of previous studies using CFD simulations, microfluidic-based models, or a full-scale artificial model of the ureter, which revealed the presence of low-velocity laminar vortices in the cavity formed by a ureteral obstruction, and their roles in promoting the attachment of bacterial cells and the deposition of encrustations [8, 9, 12, 13].

Thus, fluid flow in a tube with an abrupt change in shape is subject to significant frictional resistance, which leads to a non-uniform flow pattern and possible deposition of encrustations. The modification of critical regions, resulting in a new stent architecture, can significantly decrease the formation of encrusting deposits in patients. For example, by modifying the side-hole design in a stent, Mosayyebi et al. were able to observe a significant increase in the WSS at the inactive side-holes where encrustation was seen to occur. Such an increase in WSS can significantly decrease the formation of encrusting deposits [13]*.* Using CFD simulations, Kim et al. showed that increasing the number of side-holes can increase the drainage flow rate [16]. However, in EUO, multiple side-holes in the straight portion of the stent may be at the origin of stent obstruction [17]. Furthermore, Zheng et al. suggested that side-holes may act as initial anchoring sites for encrustation based on micro-computed tomography observations [18], and Mosayyebi et al. demonstrated that more than 60% of side-holes show low WSS using a full-scale artificial model and CFD simulations and are, thus, prone to encrustation [14]. However, although stent obstruction occurs, stent side-holes can be expected to facilitate communication between ureter and stent lumen flow. Nonetheless, the creation of “tailor-made” holes is not possible because it is impossible to predict the position and evolution of the EUO around the stent [19].

These critical regions marked by stagnant flow are not the only ones to become encrusted. In clinical settings, Amitay-Rosen et al. observed that extensive stent lumen encrustation can occur within any region of a stent and lead to stent lumen occlusion, even when the exterior stent wall is essentially free of encrusted material [20]*.* However, such extensive encrustations are not described by CFD simulations or microfluidic-based models [5, 8, 12, 13]*.* Certain approximations could be at the origin of these discrepancies. Physiologically, it is well established that the flow of urine is discontinuous, with waves of peristalsis originating in the renal pelvis and moving toward the bladder [21]*.* Previous studies have shown that physiological urodynamics are impaired in the stented ureter and the presence of a stent has been shown to cause a significant reduction in peristaltic activity. Therefore, in studies using CFD simulations, the ureter has been generally assumed to be a rigid body with steady flow [9, 14, 22]*.* However, it is possible that, even in the presence of a ureteral stent, contractions of the renal pelvis persist and that the continuous flow model is not well suited to the analysis of flow in a stented ureter.

A peristaltic movement in the ureter was solved numerically using CFD simulations by Najafi et al. They found that peristaltic motion resulted in recirculation zones developing in various parts of the ureter and, as a result, negative velocities were created, especially near the wall. Trapping and reflux phenomena were identified as the result of wall contraction [23]*.* Based on the study of Najafi et al., the present solution has been adapted to simulate discontinuous flow and the results of Fig. 3, and Fig. Online Resource 3 show irregular dissemination of the foci of encrustation depending on the ebb and flow, and appear to be closer to the clinical results observed by Amitay-Rosen et al. [20]*.* Such irregular dissemination could arise from trapping and reflux between two occluded ureteral zones. The occlusion can be pathological due to EUO or physiological due to simple concentric peristaltic contraction of the ureter.

Clearly, other circumstances influence the formation of encrusting deposits in stents. Most authors have suggested that flow processes may dictate where encrustations can grow over the stent surface [9, 12]*.* This observation can open new avenues for improving stent design via the optimization of fluid dynamics, but models based on fluid dynamics should simulate multiple instances of discontinuous flow to approximate the physiology of the ureter, even one that is stented [13]*.*

To resist protein and bacterial adhesion, innovations include modifying the ureteral stent architecture or coating the stent with antimicrobials [6, 7, 13]*.* However, several authors suggested that the stent alone introduces a relevant obstruction distributed along the entire length of the ureter [12, 16]*.* To reduce encrustation, a solution could, thus, come from reducing the thickness of the material, such as the pigtail suture stent (JFil^®^ stent) [24], or from reducing the length, such as the “Yoticurl” of Shilo et al. [25], the BraidStent^®^ of Soria et al. [26], or a customized ureteral stent of Vogt [27]. These innovative stent designs shorten or eliminate the distal part of the stent, including the bladder loop, which frequently calcifies [28] and, in any case, is not necessary for urinary flow [11, 14]*.*

Our study had several limitations. This CFD simulation solution assumed fixed placement of the stent, a uniform ureter diameter, and a constant contraction wave velocity, and did not account for the compliant nature of the ureter, the stellate-type ureteral geometry, the effect of the patient’s posture on stent movement, the chemical composition of the fluid, or the stent indwelling time. Furthermore, the bladder was modeled as an open end and the model did not account for bladder pressure or the reflux of urine from the bladder to the kidneys.

## Conclusion

The analysis of fluid dynamics through the stents tested in this study confirmed that encrustations are possible in regions where the flow is stagnant. It is preferable to favor stent models that include the smallest possible number of critical regions that favor stagnation. The discontinuous flow model provided results closer to the findings observed in human clinical practice and should be more often integrated into CFD simulations.

## Supplementary Information

Below is the link to the electronic supplementary material.

Supplementary file1 (DOCX 21 KB)

Supplementary file2 (MP4 24784 KB)

Supplementary file3 Flow vectors determined from CFD simulations near a stent side-hole for numerical models described by several authors. (a) Laminar flow model with encrusting deposits in areas with a low WSS < 1 mPa (black arrows). (b) Model as in (a) with an abruptly interrupted laminar flow to mimic pelvi-ureteric peristalsis. Encrusting deposits were observed in new discontinuous areas scattered throughout the entire external and internal surface of the stent. (c) Laminar flow model with obstruction and cavity. After temporal iterations and obtaining a steady state, vortices (V1, V2, and V3) were observed with central velocities of 10^−5^, 10^−7^, and 10^−10^ m.s^−1^, respectively. (d) Laminar flow model as in (c) with encrusting deposits in areas with a low WSS < 1 mPa. (e) Model as in (c) with an abruptly interrupted laminar flow to mimic pelvi-ureteric peristalsis. Encrusting deposits were observed in new discontinuous areas scattered throughout the entire external and internal surface of the stent (black arrows). The legends are the same as in Fig. 2. (TIF 111792 KB)

Supplementary file4 Computational mesh for calculation of the velocities and pressure in each quadrilateral element. The mesh divided the domain into 400 × 200 quadrilateral elements, in which there were 400 quadrilateral elements in the x axis and 200 in the y axis. The quadrilateral elements of the mesh are referred to using their index. The i-index refers to the quadrilateral elements in the x axis and the j-index to those in the y axis. The velocity is referred to using the velocity components u, and v in the x axis and in the y axis, respectively. The side rows and columns in grey were not used in the calculation but allowed definition of the boundary conditions. (TIF 22559 KB)

## Data Availability

All data are available in the main text or the supplementary materials.
