# Supplementary material for: Investigating the encrustation of reinforced ureteral stents by computational flow dynamic simulations
Source: World J Urol. 2023 Mar 17;41(5):1451–7. doi: 10.1007/s00345-023-04356-5 (PMC10188399; doi:10.1007/s00345-023-04356-5)
Supplement: Supplementary file 1 — Supplementary file1 (DOCX 21 KB) [file 345_2023_4356_MOESM1_ESM.docx]

**Supplementary Materials and Methods**

**The governing equations**

The working fluid in the study was assumed to be noncompressible and Newtonian. The flow was assumed to be steady and laminar (low Reynolds number of approximately 10) [1] and the volume forces $\vec{f}$ on fluid particles (gravity, inertial forces) were not considered in the study.

Temporal discretization of the Navier-Stokes equation for noncompressible flow (1) of the Materials and Methods can be written as equation (1a) in the form of nabla vectors, where $\vec{v}$, *p, η, μ,* $\vec{f}$*,t*, and Δt represent the velocity field, pressure, dynamic viscosity, density, volume forces on fluid particles, time, and time-step, respectively.

$\frac{\vec{v}^{t+1}-\vec{v}^{t}}{\Delta t}=-(\vec{v}^{t}.\vec{▽})\vec{v}^{t}-\frac{1}{\mu}\vec{▽}p^{t}+ \frac{\eta}{\mu}\vec{▽}^{2}\vec{v}^{t}+\vec{f}$ (1a)

To ensure mass conservation at a constant density, the divergence of the velocity was set to zero (no penetration of the fluid through the ureteral wall) (Equation (1b)).

$\vec{▽}. \vec{v}=$ *0* (1b)

Equation (1a) leads to scalar equations (1c) and (1d) in two dimensions, one for each velocity component (*u, v*) of $\vec{v}$.

$$\left\{ \begin{aligned} u^{t+1}=u^{t}-\Delta t\left[ \left( u^{t}\frac{\partial u^{t}}{\partial x}+v^{t}\frac{\partial u^{t}}{\partial y} \right)+\frac{1}{\mu}\frac{\partial p^{t}}{\partial x}-\frac{\eta}{\mu}\left( \frac{\partial^{2}u^{t}}{\partial x^{2}}+\frac{\partial^{2}u^{t}}{\partial y^{2}} \right) \right] (1c) \\ v^{t+1}=v^{t}-\Delta t\left[ \left( u^{t}\frac{\partial v^{t}}{\partial x}+v^{t}\frac{\partial v^{t}}{\partial y} \right)+\frac{1}{\mu}\frac{\partial p^{t}}{\partial y}-\frac{\eta}{\mu}\left( \frac{\partial^{2}v^{t}}{\partial x^{2}}+\frac{\partial^{2}v^{t}}{\partial y^{2}} \right) \right] (1d) \end{aligned} \right.$$

The terms in equations (1c) and (1d) were discretized using finite differences that approximate the derivatives using neighboring values and the second derivative using Taylor series, resulting in scalar equations (2) and (3).

$$u_{i,j}^{t+1}=u_{i,j}^{t}-u_{i,j}^{t}\frac{\Delta t}{\Delta x}\left( u_{i,j}^{t}-u_{i-1,j}^{t} \right)-v_{i,j}^{t}\frac{\Delta t}{\Delta y}\left( u_{i,j}^{t}-u_{i,j-1}^{t} \right)-\frac{\Delta t}{2\mu\Delta x}\left( p_{i+1,j}^{t}-p_{i-1,j}^{t} \right)+\frac{\eta}{\mu}\left( \frac{\Delta t}{\Delta x^{2}}\left( u_{i+1,j}^{t}+u_{i-1,j}^{t}-2u_{i,j}^{t} \right)+\frac{\Delta t}{\Delta y^{2}}\left( u_{i,j+1}^{t}+u_{i,j-1}^{t}-2u_{i,j}^{t} \right) \right) (2)$$

$$v_{i,j}^{t+1}=v_{i,j}^{t}-u_{i,j}^{t}\frac{\Delta t}{\Delta x}\left( v_{i,j}^{t}-v_{i-1,j}^{t} \right)-v_{i,j}^{t}\frac{\Delta t}{\Delta y}\left( v_{i,j}^{t}-v_{i,j-1}^{t} \right)-\frac{\Delta t}{2\mu\Delta y}\left( p_{i,j+1}^{t}-p_{i,j-1}^{t} \right)+\frac{\eta}{\mu}\left( \frac{\Delta t}{\Delta x^{2}}\left( v_{i+1,j}^{t}+v_{i-1,j}^{t}-2v_{i,j}^{t} \right)+\frac{\Delta t}{\Delta y^{2}}\left( v_{i,j+1}^{t}+v_{i,j-1}^{t}-2v_{i,j}^{t} \right) \right) (3)$$

The continuity equation for noncompressible flow does not have a dominant variable and there is no obvious way to couple velocity and pressure. Thus, equation (1a) could be derived by taking the divergence (equation (1e)) and forcing the divergence of $\vec{v}$*^t+1^* to result in zero (equation (1f)).

${\vec{▽}.\vec{v}}^{t+1}={\vec{▽}.\vec{v}}^{t}+\Delta t[-\vec{▽}.(( \vec{v}^{t}.\vec{▽})\vec{v}^{t})-\frac{1}{\mu}\vec{▽}^{2}p^{t}+ \frac{\eta}{\mu}\vec{▽}^{2}(\vec{▽}.\vec{v}^{t})]$ (1e)

$\vec{▽}^{2}p^{t}=\frac{\mu}{\Delta t}{\vec{▽}.\vec{v}}^{t}-\mu[\vec{▽}.\left( \left( \vec{v}^{t}.\vec{▽} \right)\vec{v}^{t} \right)- \frac{\eta}{\mu}\vec{▽}^{2}(\vec{▽}.\vec{v}^{t})]$ (1f)

In the study, the last two terms of the right-hand side can be ignored and equation (1f) can approximate the pressure-Poisson equation (1g) and ensure that continuity is satisfied.

$\vec{▽}^{2}p^{t}\approx\frac{\mu}{\Delta t}{\vec{▽}.\vec{v}}^{t}$ (1g)

The left-hand side terms in equation (1g) were discretized using finite differences that approximate the derivatives using neighboring values and the second derivative using Taylor series, resulting in scalar equation (1h).

$\frac{\partial^{2}p^{t}}{\partial x^{2}}+\frac{\partial^{2}p^{t}}{\partial y^{2}}\approx\frac{p_{i+1,j}^{t}+p_{i-1,j}^{t}-2p_{i,j}^{t}}{\Delta x^{2}}+\frac{p_{i,j+1}^{t}+p_{i,j-1}^{t}-2p_{i,j}^{t}}{\Delta y^{2}}$ (1h)

The right-hand side terms in equation (1h) were discretized using finite differences that approximate the derivatives using neighboring values, resulting in scalar equation (1i).

$\frac{\mu}{\Delta t}\left[ \frac{\partial u^{t}}{\partial x}+\frac{\partial v^{t}}{\partial y} \right]\approx\frac{\mu}{\Delta t}\left[ \frac{u_{i+1,j}^{t}-u_{i-1,j}^{t}}{2\Delta x}+\frac{v_{i,j+1}^{t}-v_{i,j-1}^{t}}{2\Delta y} \right]$ (1i)

Solving the right-hand side terms in equations (1h) and (1i) led to equation (4).

$$p_{i,j}^{t}=\frac{\left( p_{i+1,j}^{t}+p_{i-1,j}^{t} \right) \Delta y^{2}+\left( p_{i,j+1}^{t}+p_{i,j-1}^{t} \right) \Delta x^{2}}{2(\Delta x^{2}+\Delta y^{2})}-\frac{\mu\Delta x^{2}\Delta y^{2}}{2\left( \Delta x^{2}+\Delta y^{2} \right)}\left[ \frac{1}{\Delta t}\left( \frac{u_{i+1,j}^{t}-u_{i-1,j}^{t}}{2\Delta x}+\frac{v_{i,j+1}^{t}-v_{i,j-1}^{t}}{2\Delta y} \right) \right] (4)$$

These equations have been described by Barba et al [2] and equations (2), (3), and (4) were used in the present study for CFD simulations.

**The computational meshes**

An area of 3x3 mm² (height × width) was defined to include a transverse ureteral diameter of 3 mm [3]. This area was meshed using 200x400 quadrilateral elements as a compromise between computational cost and the robustness of the solution (corresponding to a total of 80,000 elements). The dimensions of the quadrilateral elements were 0.015x0.0075 mm² and made it possible to define the elements of the mesh using Δx and Δy, respectively (Fig. Online Resource 4).

Three meshes were designed to replicate the geometric features of a stented ureter in the presence of different types of ureteral stents (Vortek^®^ or Urosoft stents). Mesh 1 focused on the abrupt change in shape and cavity of the stent that were observed after opening it along the longitudinal axis. Mesh 2 and Mesh 3 were designed to replicate the geometric features of a stented ureter with or without an obstruction, respectively. The design for Mesh 3 was based on obstructions between the stent and ureter wall.

**Solving the governing equations**

Equation (1a) can be solved using numerical methods based on these computational meshes. A custom program in C was subsequently created to define the relevant physical models and boundary conditions and to solve the equations for the conservation of mass and momentum. A no-slip boundary condition was applied to the ureter and stent walls. The initial condition was *u*= 0, *v*= 0, and *p*= 0. For velocity, the boundary conditions were *u*= 1 mm/s at *j =*0 and *u*= 0 and *v*= 0 for the other boundary conditions (Dirichlet conditions). The imposed inlet velocity value was chosen to replicate a physiologically relevant condition, in accordance with previous studies [1,4]*.* For pressure, atmospheric pressure was applied to the device inlet and outlet and the boundary conditions were *∂p.∂x^-1^*= 0 and *∂p.∂y^-1^*= 0 near the ureter wall, the stent wall, or an obstruction (Neumann conditions). Identical boundary conditions were defined for each geometry of the stent.

The numerical simulation was started with *t =*0 and initial conditions were *η*= 0.007 Pa.s and *μ* = 1.000 kg.m^-3^. The time-step value ​​of Δt = 0.00026 s was chosen according to the Courant-Friedrichs-Lewy conditions to obtain convergence and a stable final solution. The initial conditions were used to populate the initial velocity field. The equations were used to calculate the subsequent solutions.

A steady state laminar flow model was first used to solve the governing equations. Every simulation was run for 50,000 iterations and the residual of the solution for the velocity was below 10^-9^ mm.s^-1^.

A second model with an abruptly interrupted laminar flow was used to mimic pelvi-ureteric peristalsis. Simulations of pelvic contractions were run during five periods of 10,000 iterations, each including a period at steady flow of 2,000 iterations followed by a rest period of 8,000 iterations.

The velocity gradient near the wall is called the wall shear stress (WSS) and describes the tangential force per unit area applied over a solid boundary by a ﬂuid in motion. Particular attention was devoted to the WSS and the magnitude of fluid velocity as potential determinants of the formation of encrusting deposits. In the present study, a velocity below 0.0015 mm.s^-1^ (WSS < 1 mPa) in the immediate vicinity of the stent wall was considered to be sufficiently low to lead to encrustation of the quadrilateral element. After updating the mesh with the new encrustations, the equations were used to calculate subsequent solutions until achieving a steady state with WSS > 1mPa. For each quadrilateral element, a velocity vector colored by the magnitude of the velocity in mm.s^-1^ allowed the final representation of flow in the stented ureter.

**References**

[1] Vogt B (2020) Stiffness Analysis of Reinforced Ureteral Stents Against Radial Compression: In vitro Study. Res Rep Urol 12:583–91. https://doi.org/10.2147/RRU.S285031

[2] Barba LA, Forsyth GF (2018) CFD Python: the 12 steps to Navier-Stokes equations. Journal of Open Source Education 1,21. https://doi.org/10.21105/jose.00021

[3] Clavica F, Zhao X, ElMahdy M, Drake MJ, Zhang X, Carugo D (2014) Investigating the flow dynamics in the obstructed and stented ureter by means of a biomimetic artificial model. PLoS One 9:e87433.

https://doi.org/10.1371/journal.pone.0087433

[4] De Grazia A, LuTheryn G, Meghdadi A, Mosayyebi A, Espinosa-Ortiz EJ, Gerlach R, et al (2020) A Microfluidic-Based Investigation of Bacterial Attachment in Ureteral Stents. Micromachines (Basel) 11:408. https://doi.org/10.3390/mi11040408
