# Supplementary figures and images for: Investigating the encrustation of reinforced ureteral stents by computational flow dynamic simulations
Source: World J Urol. 2023 Mar 17;41(5):1451–7. doi: 10.1007/s00345-023-04356-5 (PMC10188399; doi:10.1007/s00345-023-04356-5)

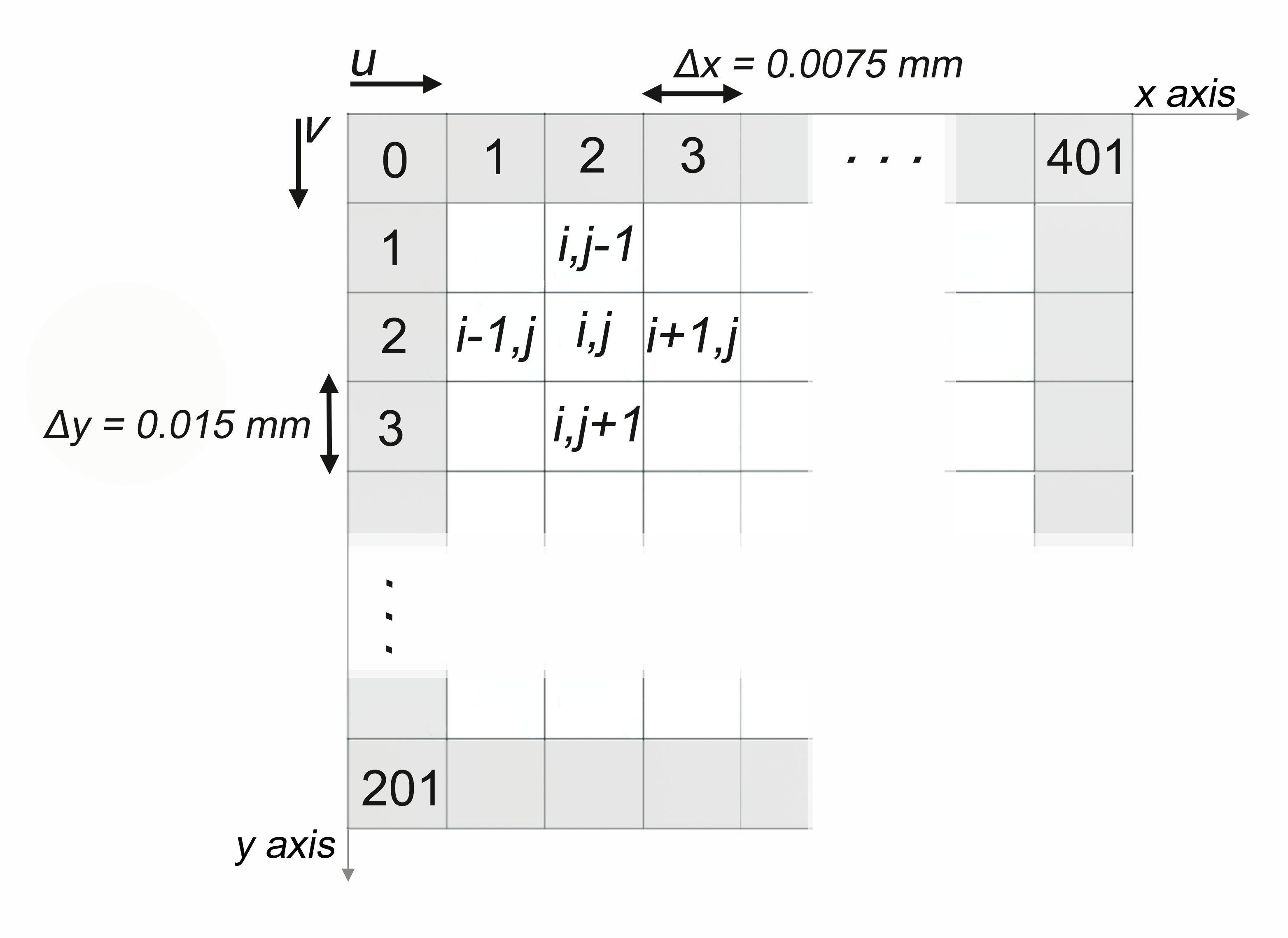

Supplement: Supplementary file 4 — Supplementary file4 Computational mesh for calculation of the velocities and pressure in each quadrilateral element. The mesh divided the domain into 400 × 200 quadrilateral elements, in which there were 400 quadrilateral elements in the x axis and 200 in the y axis. The quadrilateral elements of the mesh are referred to using their index. The i-index refers to the quadrilateral elements in the x axis and the j-index to those in the y axis. The velocity is referred to using the velocity components u, and v in the x axis and in the y axis, respectively. The side rows and columns in grey were not used in the calculation but allowed definition of the boundary conditions. (TIF 22559 KB) [file 345_2023_4356_MOESM4_ESM.tif]
